# FAMILY CAREGIVING IN THE CONTEXT OF INTENSE AND COMPLEX CARE

**DOI:** 10.1093/geroni/igz038.1646

**Published:** 2019-11-08

**Authors:** Janice Bell, Robin L Whitney, Heather M Young

**Affiliations:** 1 Betty Irene Moore School of Nursing at University of California Davis, Sacramento, California, United States; 2 Hillblom Center on Aging UCSF Fresno Medical Education Program, University of California San Francisco, Fresno, California, United States

## Abstract

Approximately four in ten family caregivers experience high intensity care, based on the number of caregiving hours, activities of daily living (ADLs) and instrumental ADLs supported for the care recipient. Using the 2015 Caregiving in the U.S. Survey, we examined outcomes associated with high (compared to low or medium) intensity of care. High intensity was positively associated with emotional stress (OR=2.10; 95%CI: 1.52-2.91); financial strain (OR=1.69; 95% CI: 1.210-2.36); physical strain (OR=3.09; 95% CI: 2.21-4.34); and declines in caregiver health (OR=2.14; 95% CI: 1.56-2.93). High intensity was also associated with greater difficulty coordinating recipient care (OR=1.96; 95%CI: 1.42-2.71), higher odds of performing complex medical/nursing tasks (OR=6.85; 95% CI: 5.27-8.90) and, among task performers, greater difficulty performing tasks (OR=2.10; 95% CI: 1.43-3.08). High intensity of care impacts caregiver health and the caregiving role in multiple domains; new clinical and policy approaches are needed to mitigate risks and ensure adequate support.

